# Prioritizing Disease Candidate Proteins in Cardiomyopathy-Specific Protein-Protein Interaction Networks Based on “Guilt by Association” Analysis

**DOI:** 10.1371/journal.pone.0071191

**Published:** 2013-08-05

**Authors:** Wan Li, Lina Chen, Weiming He, Weiguo Li, Xiaoli Qu, Binhua Liang, Qianping Gao, Chenchen Feng, Xu Jia, Yana Lv, Siya Zhang, Xia Li

**Affiliations:** 1 College of Bioinformatics Science and Technology, Harbin Medical University, Harbin, Heilongjiang Province, China; 2 Institute of Opto-electronics, Harbin Institute of Technology, Harbin, Heilongjiang Province, China; 3 National Microbiology Laboratory, Public Health Agency of Canada, Winnipeg, Manitoba, Canada; 4 Department of Cardiology, The First Affiliated Hospital of Harbin Medical University, Harbin, China; King’s College, London, United Kingdom

## Abstract

The cardiomyopathies are a group of heart muscle diseases which can be inherited (familial). Identifying potential disease-related proteins is important to understand mechanisms of cardiomyopathies. Experimental identification of cardiomyophthies is costly and labour-intensive. In contrast, bioinformatics approach has a competitive advantage over experimental method. Based on “guilt by association” analysis, we prioritized candidate proteins involving in human cardiomyopathies. We first built weighted human cardiomyopathy-specific protein-protein interaction networks for three subtypes of cardiomyopathies using the known disease proteins from Online Mendelian Inheritance in Man as seeds. We then developed a method in prioritizing disease candidate proteins to rank candidate proteins in the network based on “guilt by association” analysis. It was found that most candidate proteins with high scores shared disease-related pathways with disease seed proteins. These top ranked candidate proteins were related with the corresponding disease subtypes, and were potential disease-related proteins. Cross-validation and comparison with other methods indicated that our approach could be used for the identification of potentially novel disease proteins, which may provide insights into cardiomyopathy-related mechanisms in a more comprehensive and integrated way.

## Introduction

The cardiomyopathies are the myocardial disorders in which the heart muscle (or myocardium) is structurally and functionally abnormal, but there are not coronary artery disease, hypertension, valvular disease and congenital heart disease [1]. The cardiomyopathies can be classified into five subtypes: (*i*) hypertrophic cardiomyopathy (HCM), in which a portion of the myocardium is hypertrophied (thickened), and the heart has to work hard to pump blood [2]; (*ii*) arrhythmogenic right ventricular cardiomyopathy (ARVC), characterized by a predominant right ventricular replacement of the myocardium by partial or total adipose or fibroadipose tissue and ventricular arrhythmias [3]; (*iii*) dilated cardiomyopathy (DCM), in which the heart becomes larger (dilated), and is unable to pump blood efficiently [4]; (*iv*) restrictive cardiomyopathy (RCM), a rare form, in which the heart involves impaired diastolic filling with blood [5]; and (*v*) unclassified [6], [7]. Most cardiomyopathies are autosomal dominantly inherited. X-linked, autosomal recessive, and mitochondrial inheritance have also been reported [8]. Some environmental factors have been shown to cause cardiomyopathies, such as dietary salt exacerbates [9], abuse of alcohol, cocaine or antidepressant medications [10]. Since cardiomyopathies are major causes of morbidity and mortality and proteins are impacted by most disease-related mutations and conduct functions finally, the identification of disease-related proteins is very important for understanding mechanisms of cardiomyopathies development.

Genome-wide linkage and association studies have identified chromosomal regions which contain hundreds of candidate genes associated with these genetic diseases [11]. It still remains a big challenge to identify the potential proteins associated with genetic diseases using experimental methods with up-to-date technologies. Thus, computational predictions or candidate prioritizations of candidate proteins become attractive and draw much attention to researchers since they are cheap and effortless [12]. In recent years, identifying candidate genes of complex diseases was mainly based on biochemical networks such as metabolic networks [13], transcriptional regulatory networks [14], and protein-protein interaction (PPI) networks (PPINs) [15], which can be obtained at a large scale via high-throughput screening [16]. Several algorithms have been developed to utilize PPINs for mining or prioritizing potential disease candidate genes to understand genetic diseases [17]–[28] since the candidate genes related to specific (or similar) disease phenotypes tend to be located in a “local neighborhood” in the PPIN [29]–[31]. For example, Chen *et al.* developed a computational method to rank candidate genes for Alzheimer Disease (AD) based on an initial list of AD-related genes and public human PPI data [32]. DADA was built up as a suite to prioritize disease candidate genes accounting for the degree distribution of known disease and candidate genes, using a PPI network [33]. ToppGene and ToppNet were online candidate gene prioritization tools with high reliability based on functional similarity or network analysis in PPIN [23]. These algorithms take a set of seed proteins (genes known to be associated with the disease of interest), candidate proteins (genes in linkage intervals for the disease, genomic regions that has been associated with the disease, of interest or neighbors of seed proteins in PPINs), and a human PPIN as input. They use PPIs to infer the relationship between seed and candidate proteins, followed by ranking the candidate proteins according to the inferred relationships. Since the proteins with direct interactions tend to have the same or similar functions [34], called “guilt by association” [35], disease-related PPIN and functional similarities of protein pairs can be used to predict disease-related proteins more accurately.

In this study, we proposed a method in prioritizing disease candidate proteins to rank each protein in the network based on “guilt by association” analysis. At first, we obtained the seed proteins of DCM, HCM and ARVC from Online Mendelian Inheritance in Man (OMIM, http://www.ncbi.nlm.nih.gov/omim) [36] (other two subtypes, RCM and unclassified, were neglected since their disease genes in OMIM were very rare). We then built cardiomyopathy (DCM, HCM or ARVC)-specific PPINs composed of seed proteins and their direct neighbors (candidate proteins) from human PPI data in the STRING database [37]. Secondly, we combined the functional similarity of Gene Ontology (GO, http://www.geneontology.org/) [38] with protein interaction confidence to weigh each interacting protein pair in cardiomyopathy-specific PPINs. Subsequently, we measured the disease relevance score for each protein by adding interaction confidence and functional similarity of its neighbors and subtracting the likely effect of its interacting proteins. Finally, we took the proteins ranked at top of each candidate list in descending order of disease relevance score as potential disease-related proteins followed by leave-one-out cross-validation (LOOCV) and comparison with Chen’s protein ranking method, DADA, ToppGene and ToppNet.

## Materials and Methods

**Figure 1:**
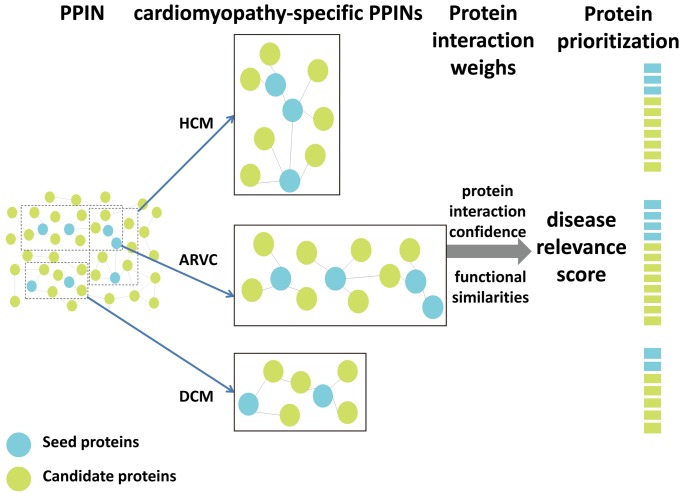
The workflow of our method in prioritizing disease candidate proteins. First, cardiomyopathy (DCM, HCM or ARVC)-specific PPINs were constructed, which were composed of seed proteins and their direct neighbors (candidate proteins) from human PPIN. Secondly, two weights (interaction confidence scores and functional similarities) were used to measure each protein interaction. The disease relevance score of each protein was measured by using these weights. Finally, the proteins ranked at top of each candidate list in descending order of disease relevance score were taken as potential disease-related proteins.

We presented a method in prioritizing disease candidate proteins to rank candidate cardiomyopathies proteins based on “guilt by association” analysis (Figure 1). Pathway enrichment analysis was then conducted to examine the relevance between the proteins at the top of each ranked list and cardiomyopathies. At last, the proposed method was compared with other methods to test its performance.

### Screening of Seed Proteins of Cardiomyopathies

The disease-related genes of three subtypes of cardiomyopathy were obtained from OMIM. As a result, 33 DCM, 24 HCM, and 9 ARVC genes were selected as seed genes, respectively. These genes were further converted into their corresponding standard symbols by using the HUGO Gene Nomenclature Committee (HGNC) database (http://www.genenames.org) [39] (Table 1) and seed proteins were generated as referred to the proteins corresponding to these seed genes.

**Table 1:** Official symbols of seed genes of DCM, HCM and ARVC.

	DCM	HCM	ARVC
Seed genes/	NEXN (Q0ZGT2*)	NEXN (Q0ZGT2)	RYR2 (Q92736)
proteins	LMNA (P02545)	TNNT2 (P45379)	TMEM43 (Q9BTV4)
	TNNT2 (P45379)	TTN (Q8WZ42)	RPSA (P08865)
	PSEN2 (P49810)	CAV3 (P56539)	DSP (P15924)
	ACTN2 (P35609)	MYL3 (P08590)	PKP2 (Q99959)
	TTN (Q8WZ42)	TNNC1 (P63316)	TGFB3 (P10600)
	DES (P17661)	MYOZ2 (Q9NPC6)	JUP (P14923)
	SCN5A (Q14524)	SLC25A4 (P12235)	DSC2 (Q02487)
	TNNC1 (P63316)	MYO6 (Q9UM54)	DSG2 (Q14126)
	SDHA (P31040)	PLN (P26678)	
	SGCD (Q92629)	PRKAG2 (Q9UGJ0)	
	DSP (P15924)	VCL (P18206)	
	PLN (P26678)	COX15 (Q7KZN9)	
	EYA4 (O95677)	CSRP3 (P50461)	
	GATAD1 (Q8WUU5)	MYBPC3 (Q14896)	
	FKTN (O75072)	MYL2 (P10916)	
	VCL (P18206)	MYH6 (P13533	
	LDB3 (O75112)	MYH7 (P12883)	
	RBM20 (Q5T481)	ACTC1 (P68032)	
	BAG3 (O95817)	TPM1 (P09493)	
	CSRP3 (P50461)	CALR3 (Q96L12)	
	MYBPC3 (Q14896)	TNNI3 (P19429)	
	ABCC9 (O60706)	MYLK2 (Q9H1R3)	
	TMPO (P42166)	JPH2 (Q9BR39)	
	MYH6 (P13533)		
	MYH7 (P12883)		
	PSEN1 (P49768)		
	ACTC1 (P68032)		
	TPM1 (P09493)		
	TCAP (O15273)		
	DSG2 (Q14126)		
	TNNI3 (P19429)		
	DMD (P11532)		

* Accession number of the corresponding protein.

### Construction of Weighted Cardiomyopathy-specific PPINs

The method of the nearest-neighbor expansion was applied to obtain the direct neighbors of seed proteins of DCM, HCM and ARVC from human PPI data in the STRING database [37] (version 8.3). To be more comprehensive, all the interaction relationships of seed proteins were kept as original ones from STRING. DCM, HCM and ARVC-specific PPINs were built. As a result, 5624, 3869, 2173 nodes and 14569, 8972, 3003 edges were generated, respectively. Direct neighbors of seed proteins in these cardiomyopathy-specific PPINs were considered as the candidate proteins of three cardiomyopathy subtypes.

Two weights were used to measure each protein interaction (*i.e.* each edge of the network). The first weight is the confidence score 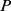 from STRING [40], and the second one is the functional similarity 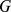 by combining functional enrichment analysis of GO. The functional similarity 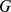 was computed by employing an R package GOSim [41], which ranged from 0 to 1 according to GO annotations. Finally, the edge-weighted cardiomyopathy (DCM, HCM or ARVC)-specific PPINs were constructed.

### Calculation of Disease Relevance Score Based on “Guilt by Association” Analysis

We presented a method in prioritizing disease candidate proteins to measure the relevance of each candidate protein to a disease in each cardiomyopathy-specific PPIN. In this method, the relevance between a protein and seed proteins in its neighborhood was estimated by “guilt by association” effects of seed proteins to candidate proteins, *i.e.* connectivity, interaction confidences and functional similarities of each protein in cardiomyopathy-specific PPINs. Briefly, the disease relevance score of one protein was measured by adding interaction confidence and functional similarity of its neighbors and by subtracting the effect of promiscuous connections between this protein and its interacting proteins. A disease relevance score 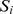 for each protein 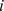 in each cardiomyopathy-specific PPIN was calculated as follows:
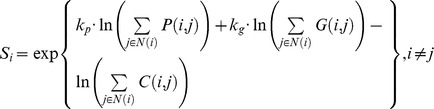
where 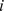 and 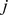 represent two proteins; 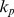 and 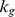 are empirical constants (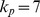 and 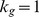 were set up after screening them from all combinations of 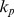 and 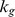 from 1 to 10, respectively. LOOCV was used to identify constants and to reduce overfit bias); 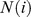 is the set of proteins interacting with 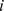 in the cardiomyopathy-specific PPIN; 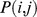 is the interaction confidence score of protein pair 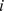 and *j*; 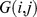 is the functional similarity value of protein pair 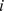 and *j*; and 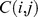 is 1 if protein *j* belongs to the cardiomyopathy-specific PPIN (or 0 otherwise). The score 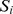 ranks higher in the situations where there are more interacting proteins, higher confidence interactions and functional similarities with seed proteins among its neighbors.

Each candidate protein in the network was ranked by descending order of 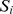, and the performance of these prioritizations was assessed.

### Pathway Analysis of Top Ranked Candidate Proteins

To further examine the functional relevance between the candidate proteins that were ranked at the top of each ranked list and cardiomyopathies, KEGG pathway enrichment analysis was applied for top 50 candidate proteins using the Functional Annotation Tool in DAVID Bioinformatics Resources 6.7 (http://david.abcc.ncifcrf.gov/) [42], [43]. P value less than 0.05 was considered as significance.

### Assessment of the Developed Method Prioritizing Disease Candidate Proteins

LOOCV was applied to assess the developed method. For all seed proteins, one protein was removed as a test protein at each time, and was added to candidate proteins. Cardiomyopathy-specific PPINs were then reconstructed using the newly generated sets of seed proteins and their direct neighbors. All the candidate proteins were ranked by the developed method to determine the rank of the test protein. This procedure was repeated until all the seed proteins were used up as test proteins. In the end, the result generated by our method was compared with those of Chen’s protein ranking method, DADA, ToppGene and ToppNet using the same seed and candidate proteins as our method did.

To compare these methods, receiver operating characteristic (ROC) curves were plotted by sensitivity and specificity values of prioritizations. Sensitivity refers to the percentage of the removed seed proteins which were ranked over a particular threshold. Specificity refers to the proportion of non-test proteins which were ranked below the threshold [44]. The area under curve (AUC) is a standard measure of performances of these methods.

At last, we analyzed the top 50 candidate proteins obtained by our method and compared them to ones obtained from Chen’s protein ranking method, which had the best performance among the methods used to compare with our method, to further assess the performance of our method by literature review from the PubMed database.

## Results

### Prioritizations of Cardiomyopathies’ Candidate Proteins Based on “Guilt by Association” Analysis

We used our developed method to calculate disease relevance scores of all candidate proteins and rank them in each of cardiomyopathy-specific PPINs. To examine the effectiveness of our disease relevance scores in ranking disease proteins, scores of seed proteins were also calculated, and compared with those of candidate proteins. It was found that scores of all seed proteins were larger than those of candidate proteins. We then focused on the relevance between candidate proteins ranked at the top of the ranked list and the corresponding disease. Top 50 DCM candidate proteins were identified and listed in Table 2.

**Table 2:** Top 50 candidate proteins from DCM-specific PPIN.

Protein*	Accession number	Rank	Disease relevance score	Relevance	Literature
MYL2	P10916	1	107629670.500	cardiomyopathy	[54], [55]
MYL3	P08590	2	73161485.690	cardiomyopathy	[54], [55]
TNNI1	P19237	3	52203517.710		
MYH14	Q7Z406	4	45310956.140		
NEB	P20929	5	44470968.060		
TNNI2	P48788	6	29830367.350		
GJA1	P17302	7	24199871.590	cardiac arrhythmias	[56]
ACTA1	P68133	8	23568069.020	DCM	[45]
MYL1	P05976	9	19532022.970		
TNNC2	P02585	10	18200557.600		
TPM2	P07951	11	17743459.410	cardiac dysfunction	[57]
VIM	P08670	12	15069691.590		
TNNT3	P45378	13	12691030.660		
TNNT1	P13805	14	10598443.490		
GJA5	P36382	15	9497687.715	cardiac arrhythmias	[56]
SP4	Q02446	16	7947705.183		
MYL4	P12829	17	7586555.815		
TPM3	P06753	18	7337310.387		
TMOD1	P28289	19	7014582.495	DCM	[46]
MYOT	Q9UBF9	20	6610783.862		
TPM4	P67936	21	6600584.576		
MYH3	P11055	22	6368705.641		
MYBPC1	Q00872	23	6317707.232		
MYBPC2	Q14324	24	4820328.071		
CAV3	P56539	25	3746694.232	DCM	[47], [48]
MYOD1	P15172	26	2449472.494	cardiomyopathy	[78]
CALM1	P62158	27	2291170.029	DCM	[45]
ACTB	P60709	28	2039574.700		
MYOG	P15173	29	1304602.403		
DNAH8	Q96JB1	30	1247401.222		
CKM	P06732	31	1139350.347	DCM	[49]
CAPN3	P20807	32	1108281.072		
PRKAG2	Q9UGJ0	33	1068188.085	cardiomyopathy	[79]
ZMPSTE24	O75844	34	1050441.814	DCM	[50]
AMY1A	P04745	35	1028615.159		
AMY1B	P04745	36	983506.614		
HRAS	P01112	37	931783.319	cardiomyopathy	[80]
HLA-DR4	P13760	38	926276.147	DCM	[51], [52]
DNM2	P50570	39	896713.915		
NKX2-5	P52952	40	791481.623	cardiomyopathy	[81]
FXN	Q16595	41	591821.092	cardiomyopathy	[82]
DYSF	O75923	42	591430.047	DCM	[53]
AMY2A	P04746	43	547354.552		
ACTG1	P63261	44	532313.791		
C1QBP	Q07021	45	491410.248	cardiac cell damage	[58]
CALD1	Q05682	46	485082.005		
AMY2B	P19961	47	476690.079		
DAG1	Q14118	48	475398.858	DCM	[59], [60]
AMY1C	P04745	49	455785.472		
PRKCA	P17252	50	442473.436		

* Proteins are represented in their corresponding gene symbols.

In these candidate proteins, 9 out of 50 have been reported to be DCM-related proteins (as shown in Table 2) in literature using the PubMed database. For example, TMPP could be a promising drug for prevention and treatment of DCM since it reduces expression of ACTA1 and CALM1 in the DCM heart [45]. TMOD1 was shown to be over-expressed and associated with DCM in juvenile mice [46]. CAV3 was found to be mutated in two patients with DCM [47], [48]. Protein levels of CKM activity were found to decrease in DCM patients [49]. Histopathological analysis of the mutant mice with disruption of the gene ZMPSTE24 revealed DCM [50]. Statistically elevated frequency of HLA-DR4 allele was found in patients with DCM compared with ones in controls [51], [52]. DYSF generally resulted in mild cardiac abnormalities to severe DCM [53]. The rest of our potential disease proteins (41) were not directly associated to DCM in literature review. However, 7 of them may be related to the processes associated with DCM since these candidate proteins were directly associated to other types of cardiomyopathies, such as HCM (*e.g.* MYL2 and MYL3 [54], [55]). Four proteins (GJA1, TPM2, GJA5 and C1QBP) were related with cardiac arrhythmias, cardiac dysfunction and cardiac cell damage, and might also be responsible for DCM [56]–[58]. More experiments are needed to study their associations with DCM.

For HCM-specific PPIN and ARVC-specific PPIN, top 50 candidate proteins were identified and their relevance with cardiomyopathies was listed in Table S1 and S2, respectively. It demonstrated that the most of top 50 candidate proteins identified in our method were associated with cardiomyopathies (Table 2, Table S1, and S2).

**Figure 2:**
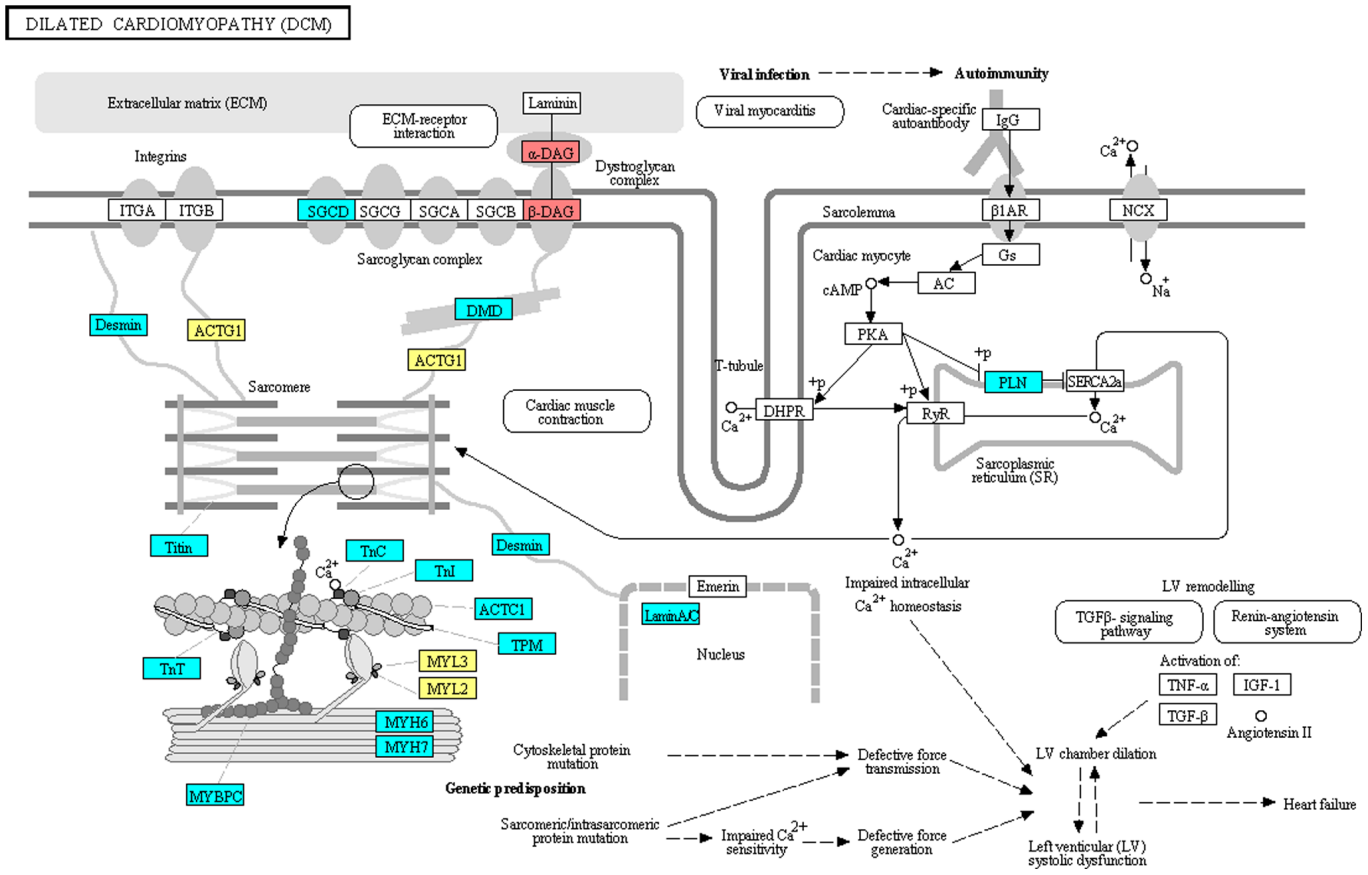
DCM pathway. DCM seed proteins are colored in cyan. Red nodes are proteins which were verified to be DCM-related proteins, and yellow nodes represent proteins which are potential DCM-related proteins.

We searched cardiomyopathy seed proteins and top 50 candidate proteins in their corresponding disease pathways in KEGG. Four candidate proteins were found in DCM pathway (Figure 2), one of which (DAG1) have been validated to be a DCM-related protein in an investigation of glycosylation pathways in biopsied heart tissue due to autosomal recessive mutations [59], [60].

Although MYL2 and MYL3 have not been validated to be directly associated to DCM, their relationships with other cardiomyopathies have been mentioned in literature. They encode sarcomere proteins that cause adult-onset cardiomyopathies when mutated [54], [55], and may cause DCM eventually.

ACTG1 was shown to link to the seed gene DMD and sarcomere, two important factors of DCM in the DCM pathway [61], [62]. There is no evidence about the relationship between ACTG1 and cardiomyopathies and more studies are needed.

Moreover, five and seven candidate proteins in HCM and ARVC pathways were found, respectively. One from each pathway has been validated to be disease proteins, respectively (Figure S1 and S2). These results demonstrated that our method in prioritizing disease candidate proteins could provide a new alternative for researchers to predict novel disease proteins, *i.e.* the top ranked candidate proteins without literature review.

### Pathway Analysis of the Top Ranked Candidate Proteins

**Figure 3:**
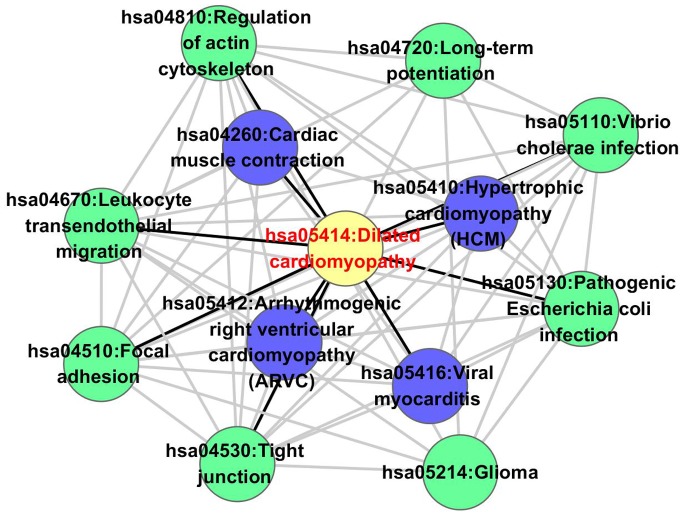
DCM pathway and its relevant pathways. DCM pathway is colored in yellow. Purple nodes are DCM-related pathways, and green nodes are other pathways. Black edges connect pathways which are directly connected to the DCM pathway.

KEGG pathway enrichment analysis (p<0.05) was performed for the top 50 candidate proteins to illustrate the relationships between disease pathways of three subtypes of cardiomyopathies and other pathways (Figure 3, Figure S3, and S4). It was shown that DCM disease pathway was related to both HCM and ARVC pathways. DCM-related pathways that DCM seed genes enriched in were in the inner space (Figure 3).

We surveyed relationships between the DCM pathway and other pathways using literature from the PubMed database. It was found that reduced focal adhesion kinase-S910 phosphorylation might contribute to sarcomere disorganization in DCM [63], suggesting direct regulation of focal adhesion to DCM. STC1, which was up-regulated in DCM, effectively blocked down-regulation of endothelial tight junction proteins at both mRNA and protein levels [64]. Moreover, deregulation of proteins of carbohydrate metabolism, the actin cytoskeleton, and extracellular matrix remodeling were observed in DCM patients [65]. The association between Escherichia coli infection and DCM was established in a patient who was diagnosed as DCM after the onset of hemolytic uremic syndrome caused by pathogenic Escherichia coli infection [66]. In addition, leukocyte transendothelial migration was found to be pivotal to the inflammatory response [67], which could lead to direct injury or severe host disease, such as DCM [68].

### Assessment of the Developed Method

To assess the performance of our method, we compared our method with Chen’s protein ranking method, DADA, ToppGene and ToppNet using LOOCV. ROC curves were then plotted to demonstrate performances of these methods for DCM. It was found that our method reached a higher AUC score (0.963) than Chen’s protein ranking method (0.956), DADA (0.854), ToppGene (0.884) and ToppNet (0.741), indicating that our method was more sensitive and specific in ranking the test proteins. The performances of these five methods were also compared for HCM and ARVC, and similar results were obtained (Table 3), except that DADA was better only for HCM. These results demonstrated that our method had a better overall performance.

**Table 3:** AUC for three subtypes of cardiomyopathies obtained using five different methods.

	Our developed method	Chen’s protein ranking method	DADA	ToppGene	ToppNet
DCM	0.963	0.956	0.854	0.884	0.741
HCM	0.919	0.916	0.979	0.911	0.716
ARVC	0.995	0.934	0.770	0.946	0.756

**Figure 4:**
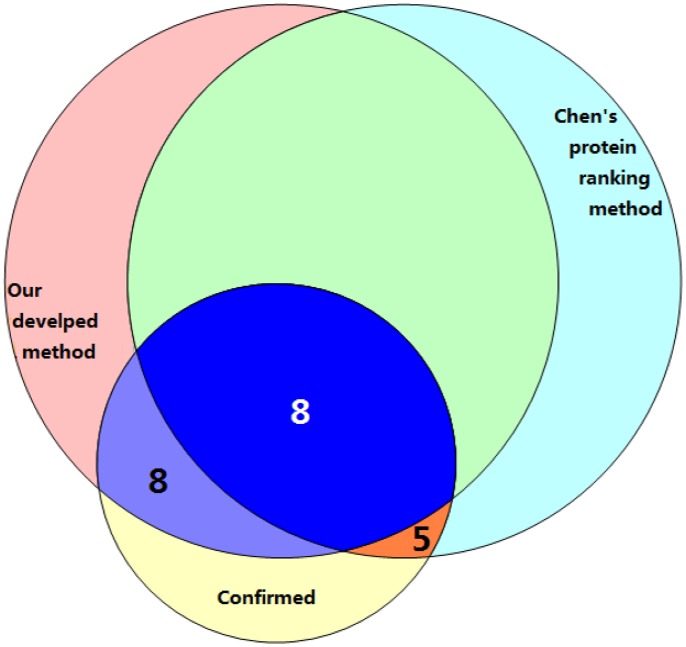
The number of proteins related with DCM. 50 potential disease proteins identified either by our developed method (the top left circle), or by Chen’s protein ranking method (the top right circle), and the number of proteins which have been confirmed to be related with DCM in literature were plotted.

To further test the performance of our method, top 50 candidate proteins from our method were compared with those from Chen’s protein ranking method by literature review. The result for DCM was shown in Figure 4. We found 36 proteins in both protein sets, in which 8 were confirmed to be DCM-related (Table 2). Among the remaining proteins, 8 in our potential protein list (Table 2) and 5 in Chen’s protein ranking list [69]–[73] were found to be related to the processes associated with DCM.

Results for HCM and ARVC were shown in Figure S5 and S6. In general, top 50 candidate proteins from our method contained more cardiomyopathy-related proteins than those from Chen’s protein ranking method.

### Crosstalk of Three Subtypes of Cardiomyopathies

To illustrate relationship among three subtypes of cardiomyopathies, we compared seed proteins of DCM, HCM and ARVC. Thirteen seed proteins were found in both DCM and HCM, suggesting that there might be similar mechanisms in these diseases [74]. For example, most of these proteins comprised the sarcomeric proteins (Figure 2 and Figure S1). However, there were no common seed genes identified to be shared between ARVC and DCM or HCM.

To further explore relationship among three subtypes of cardiomyopathies, we compared the top 50 candidate proteins of each cardiomyopathy. Only 2 proteins were found in the protein lists of all the three cardiomyopathies and 29 proteins were found in those of both DCM and HCM. Since all the pathways of these subtypes of cardiomyopathies involve integrins, dystroglycan complex and sarcomere (Figure 2, Figure S1, and S2), which are important in cardiac myocyte and cardiac muscle contraction [75], [76], it suggested that there was a crosstalk of these three subtypes of cardiomyopathies, especially between DCM and HCM.

Investigation of disease pathways and their related pathways (Figure 3, Figure S3, and S4) showed that these three subtypes of cardiomyopathies were also closely related. Four pathways were found to share among the three diseases: Tight junction, Focal adhesion, Regulation of actin cytoskeleton and Leukocyte transendothelial migration. These results further implied a crosstalk of three subtypes of cardiomyopathies.

## Discussion

In our study, OMIM was applied to obtain seed proteins of three subtypes of cardiomyopathies, DCM, HCM and ARVC. With these seed proteins, we built cardiomyopathy (DCM, HCM or ARVC)-specific PPINs through the nearest-neighbor expansion method and weighted each protein pair in the network. We then developed a method to prioritize disease candidate proteins by calculating disease relevance score and ranking each protein in the network. As a result, it was shown that proteins ranked at the top of candidate proteins were potential cardiomyopathy-related proteins, which opened a new door for the further research of cardiomyopathy pathogenesis. By analyzing top 50 candidate proteins, it was found that most of them and cardiomyopathy seed proteins shared common disease-related KEGG pathways. The performance of our method was evaluated based on the following criteria: *1)*. literature review. By the literature review from the PubMed database, it was found that top 50 candidate proteins were closely correlated with cardiomyopathies; *2)*. KEGG pathway enrichment analysis. Our results revealed that the most of seed and top 50 candidate proteins were enriched in disease-related functional classes and pathways; *3).* leave-one-out cross-validation. In this validation, candidate proteins were ranked by using our method in prioritizing disease candidate proteins, Chen’s method, DADA, ToppGene and ToppNet. It was found that the overall performance of our method was better than all the others.

The reliability and precision of our method were improved based on the following factors. Firstly, we measured the relevance of each protein with disease based on “guilt by association” analysis by adding interaction confidence and functional similarity of its neighbors and subtracting the effect of promiscuously connections between the protein and its interacting proteins. Secondly, the relationships of comprehensive human disease protein interaction were obtained using protein interaction data from the STRING database. Thirdly, the establishment of cardiomyopathy-specific PPINs using disease-related seed proteins enhanced specific connections between disease proteins and their interacting proteins, reduced promiscuous connections, and had higher fidelity interaction confidence. Finally, identified top 50 candidate proteins in weighted cardiomyopathy-specific PPINs by using our method in prioritizing disease candidate proteins were more strongly associated with cardiomyopathies, and the crosstalk of three subtypes of cardiomyopathies could be obtained through common proteins in three ranked lists. As a result, we obtained more proteins which were closely associated with cardiomyopathies in literature and some new proteins with the unknown roles involving in cardiomyopathies ranked at the top of candidate proteins. Further investigation is required to prove their disease relevance.

To further examine the performance of our method in prioritizing disease candidate proteins, we compared it with GeneMANIA [77] and ToppGenet in ToppGene. GeneMANIA and ToppGenet prioritize neighboring proteins of seeds in their background networks, which were different from our method. It was shown that our method obtained a higher AUC score than other two methods (Table 4) for each subtype of cardiomyopathies. It is worth noting that better AUC scores were obtained using ToppGenet with distance 3 or 4 from seeds, which implied that proteins not in the direct neighborhood of seed proteins might also be disease-related. Besides, since our method depends on PPI, disease proteins with unknown PPI failed to be identified or ranked. We will develop a more comprehensive approach taking proteins not in the direct neighborhood of seed proteins and other information, such as expression, into account in the future to predict disease associated proteins.

**Table 4:** AUC for three subtypes of cardiomyopathies obtained using GeneMANIA and ToppGenet.

	Our developed method	GeneMANIA	ToppGenet				
				Distance to seeds			
				1	2	3	4
DCM	0.963	0.466	Network based	0.373	0.660	0.728	0.725
			Functional annotation based	0.485	0.724	0.774	0.809
HCM	0.919	0.588	Network based	0.291	0.670	0.767	0.776
			Functional annotation based	0.369	0.834	0.905	0.904
ARVC	0.995	0.569	Network based	0.519	0.630	0.665	0.669
			Functional annotation based	0.801	0.873	0.894	0.894

In conclusion, the method in prioritizing disease candidate proteins based on “guilt by association” analysis has proven its ability to more precisely identify potential disease-related proteins. This study not only provided a new methodology for studying human cardiomyopathy disease, but also shed light on process and mechanisms of human cardiomyopathy and other complex diseases.

Full names of all gene/protein symbols used in the main text are listed below:

ACTA1: actin, alpha 1, skeletal muscle

CALM1: calmodulin 1

TMOD1: tropomodulin 1

CAV3: caveolin 3

CKM: creatine kinase, muscle

ZMPSTE24: zinc metallopeptidase STE24

HLA-DR4: major histocompatibility complex, class II, DR beta 1

DYSF: dysferlin, limb girdle muscular dystrophy 2B

MYL2: myosin, light chain 2, regulatory, cardiac, slow

MYL3: myosin, light chain 3, alkali; ventricular, skeletal, slow

DAG1: dystroglycan 1

ACTG1: actin, gamma 1

DMD: dystrophin

STC1: stanniocalcin 1

## Supporting Information

Figure S1 **HCM pathway.** HCM seed proteins are colored in cyan. Red nodes are proteins which were verified to be HCM-related proteins, and yellow nodes represent proteins which are potential HCM-related proteins.
(DOC)

Figure S2 **ARVC pathway.** ARVC seed proteins are colored in cyan. Red nodes are proteins which were verified to be ARVC-related proteins, and yellow nodes represent proteins which are potential ARVC-related proteins.
(DOC)

Figure S3 **HCM pathway and its relevant pathways.** The HCM pathway is colored in yellow. Purple nodes are HCM-related pathways, and green nodes are other pathways. Black edges connect pathways which are directly connected to the HCM pathway.
(DOC)

Figure S4 **ARVC pathway and its relevant pathways.** The ARVC pathway is colored in yellow. Green nodes are other pathways. Black edges connect pathways which are directly connected to the ARVC pathway.
(DOC)

Figure S5 **The number of proteins related with HCM.** 50 potential disease proteins identified either by our developed method (the top left circle) or by Chen’s protein ranking method (the top right circle), and the number of proteins that have been confirmed to be related with HCM in literature were plotted.
(DOC)

Figure S6 **The number of proteins related with ARVC.** 50 potential disease proteins identified either by our developed method (the top left circle) or by Chen’s protein ranking method (the top right circle), and the number of proteins which have been confirmed to be related with ARVC in literature were plotted.
(DOC)

Table S1 **Top 50 candidate proteins from HCM-specific PPIN.**
(DOC)

Table S2 **Top 50 candidate proteins from ARVC-specific PPIN.**
(DOC)
