# Supplementary material for: Prioritizing Disease Candidate Proteins in Cardiomyopathy-Specific Protein-Protein Interaction Networks Based on “Guilt by Association” Analysis
Source: PLoS One. 2013 Aug 5;8(8):e71191. doi: 10.1371/journal.pone.0071191 (PMC3733802; doi:10.1371/journal.pone.0071191)

**Figure S1. HCM pathway.**

HCM seed proteins are colored in cyan. Red nodes are proteins which were verified to be HCM-related proteins, and yellow nodes represent proteins which are potential HCM-related proteins.


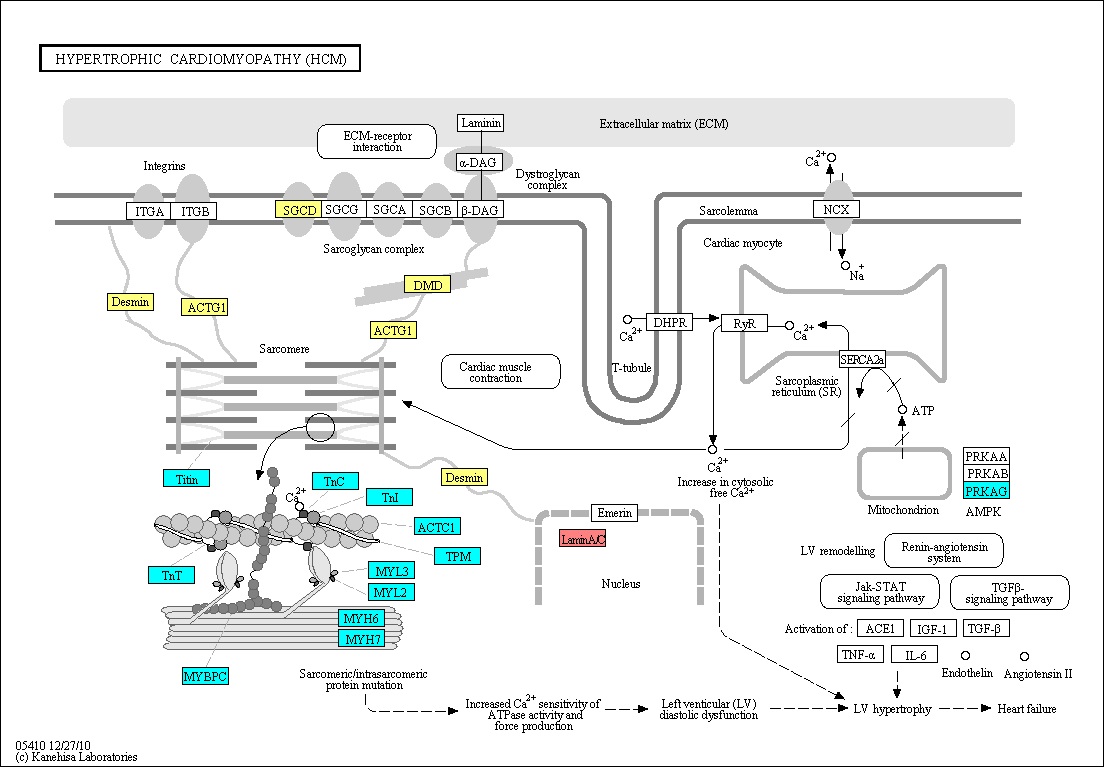

Supplement: Figure S1 — HCM pathway. HCM seed proteins are colored in cyan. Red nodes are proteins which were verified to be HCM-related proteins, and yellow nodes represent proteins which are potential HCM-related proteins. (DOC) [file pone.0071191.s001.doc]
