# Supplementary material for: Prioritizing Disease Candidate Proteins in Cardiomyopathy-Specific Protein-Protein Interaction Networks Based on “Guilt by Association” Analysis
Source: PLoS One. 2013 Aug 5;8(8):e71191. doi: 10.1371/journal.pone.0071191 (PMC3733802; doi:10.1371/journal.pone.0071191)

**Figure S2. ARVC pathway.**

ARVC seed proteins are colored in cyan. Red nodes are proteins which were verified to be ARVC-related proteins, and yellow nodes represent proteins which are potential ARVC-related proteins.


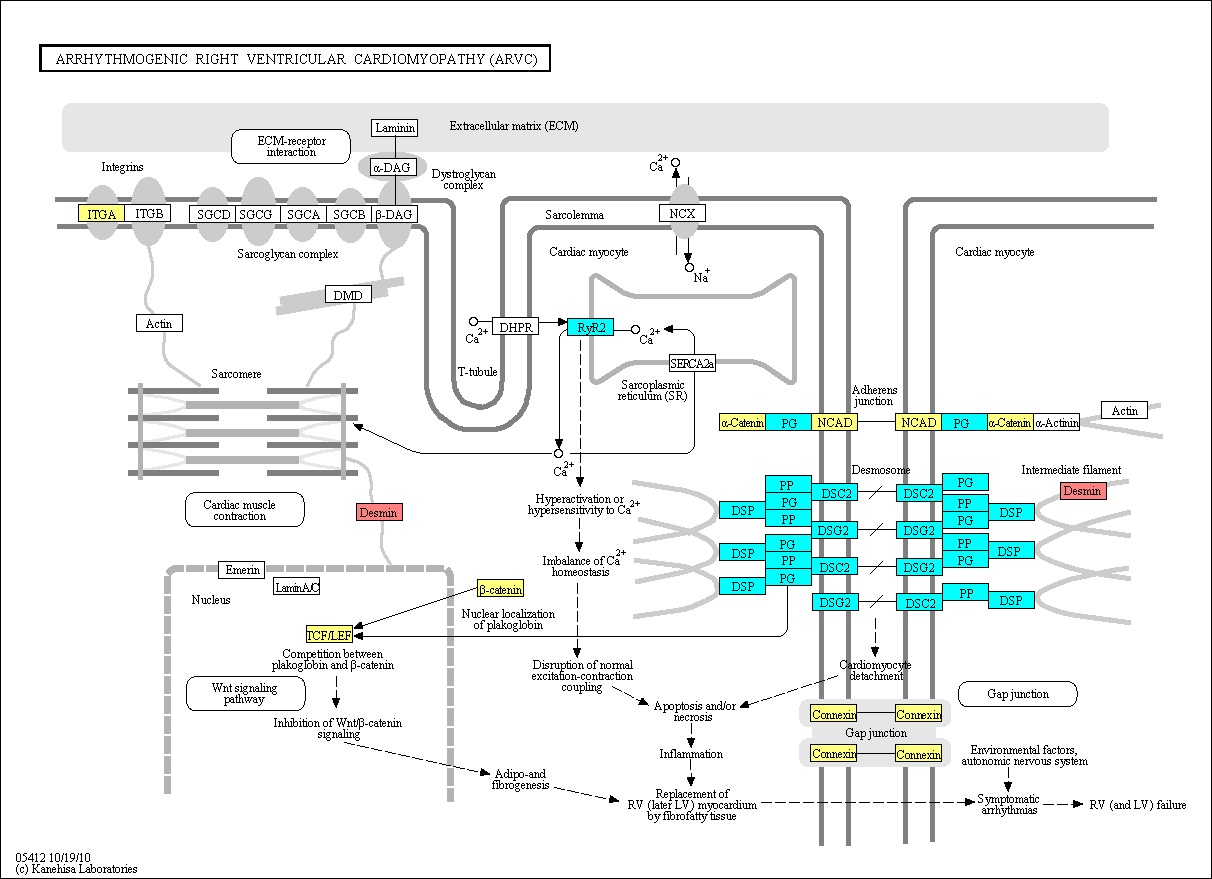

Supplement: Figure S2 — ARVC pathway. ARVC seed proteins are colored in cyan. Red nodes are proteins which were verified to be ARVC-related proteins, and yellow nodes represent proteins which are potential ARVC-related proteins. (DOC) [file pone.0071191.s002.doc]
