# Supplementary material for: Prioritizing Disease Candidate Proteins in Cardiomyopathy-Specific Protein-Protein Interaction Networks Based on “Guilt by Association” Analysis
Source: PLoS One. 2013 Aug 5;8(8):e71191. doi: 10.1371/journal.pone.0071191 (PMC3733802; doi:10.1371/journal.pone.0071191)

**Figure S3. HCM pathway and its relevant pathways.**

The HCM pathway is colored in yellow. Purple nodes are HCM-related pathways, and green nodes are other pathways. Black edges connect pathways which are directly connected to the HCM pathway.


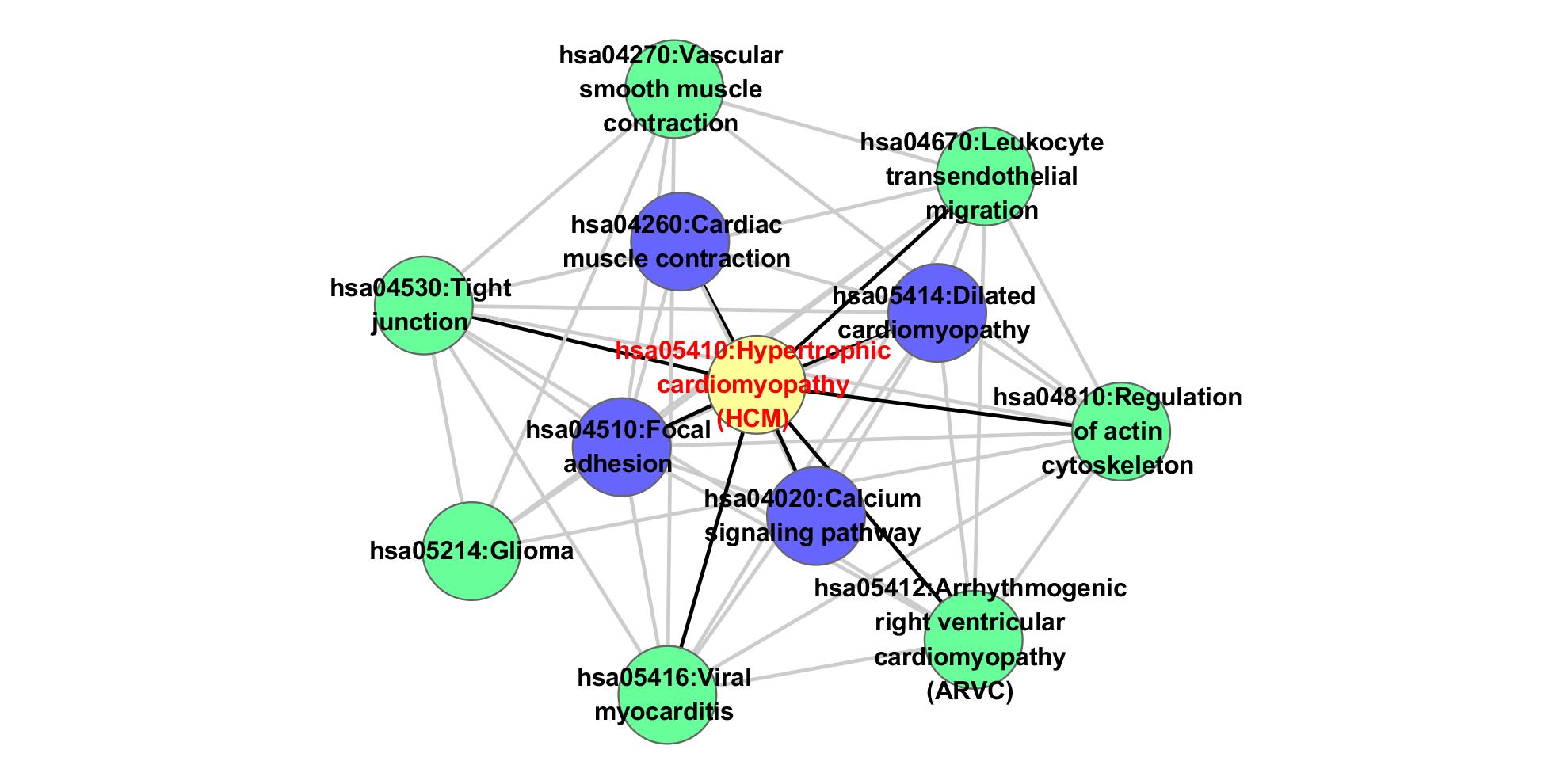

Supplement: Figure S3 — HCM pathway and its relevant pathways. The HCM pathway is colored in yellow. Purple nodes are HCM-related pathways, and green nodes are other pathways. Black edges connect pathways which are directly connected to the HCM pathway. (DOC) [file pone.0071191.s003.doc]
