# Supplementary material for: Prioritizing Disease Candidate Proteins in Cardiomyopathy-Specific Protein-Protein Interaction Networks Based on “Guilt by Association” Analysis
Source: PLoS One. 2013 Aug 5;8(8):e71191. doi: 10.1371/journal.pone.0071191 (PMC3733802; doi:10.1371/journal.pone.0071191)

**Figure S4. ARVC pathway and its relevant pathways.**

The ARVC pathway is colored in yellow. Green nodes are other pathways. Black edges connect pathways which are directly connected to the ARVC pathway.


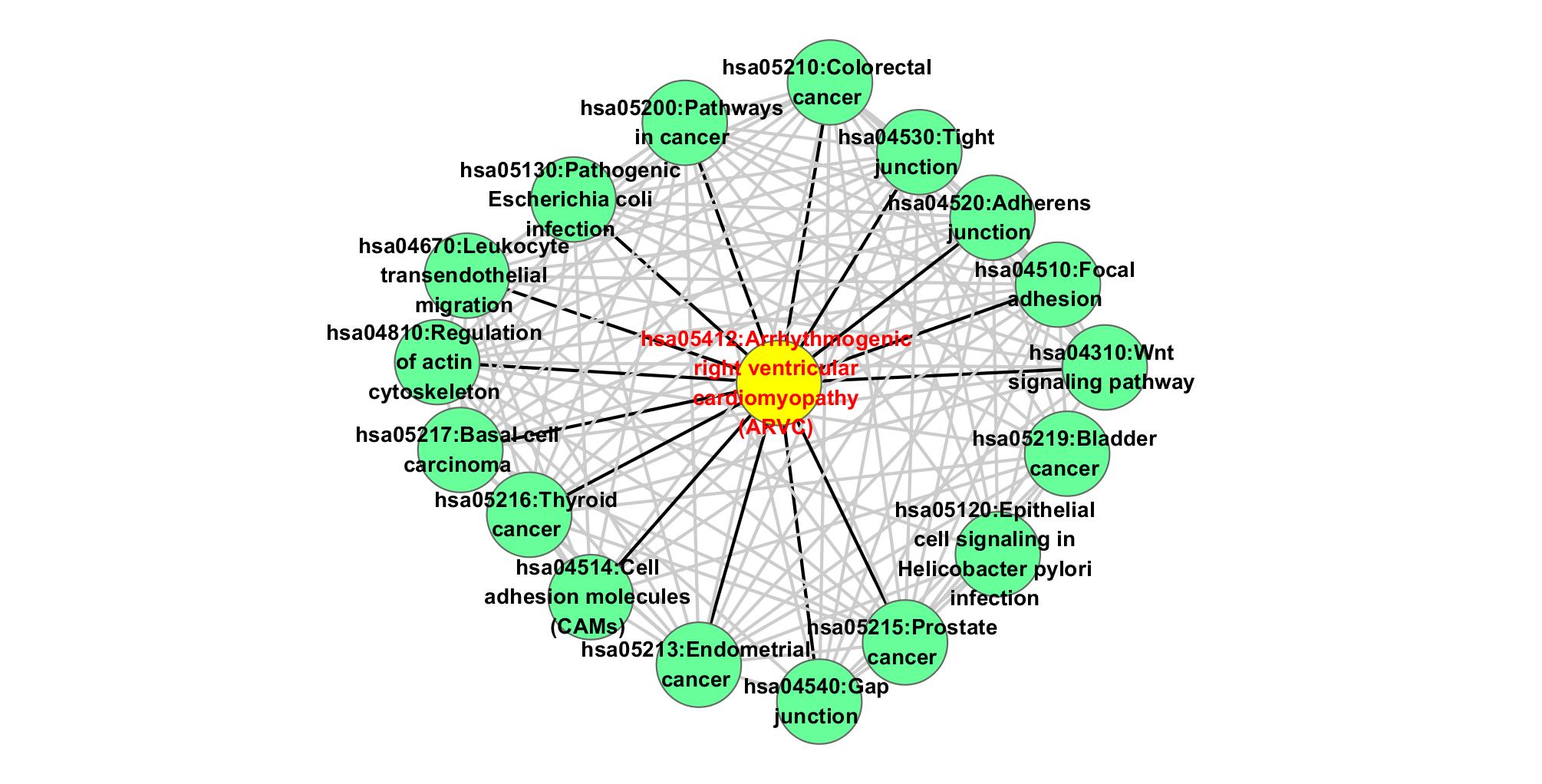

Supplement: Figure S4 — ARVC pathway and its relevant pathways. The ARVC pathway is colored in yellow. Green nodes are other pathways. Black edges connect pathways which are directly connected to the ARVC pathway. (DOC) [file pone.0071191.s004.doc]
