# Supplementary material for: Prioritizing Disease Candidate Proteins in Cardiomyopathy-Specific Protein-Protein Interaction Networks Based on “Guilt by Association” Analysis
Source: PLoS One. 2013 Aug 5;8(8):e71191. doi: 10.1371/journal.pone.0071191 (PMC3733802; doi:10.1371/journal.pone.0071191)

**Figure S6. The number of proteins related with ARVC.**

50 potential disease proteins identified either by our developed method (the top left circle) or by Chen's protein ranking method (the top right circle), and the number of proteins which have been confirmed to be related with ARVC in literature were plotted.


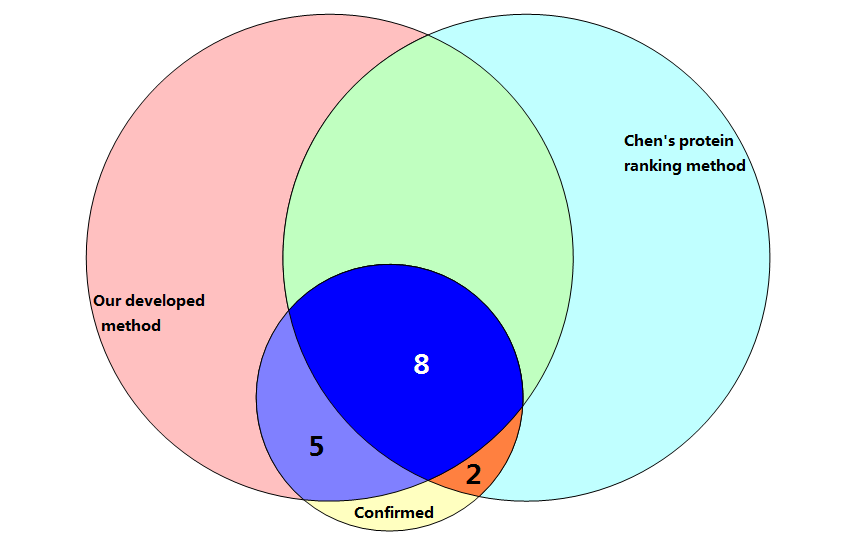

Supplement: Figure S6 — The number of proteins related with ARVC. 50 potential disease proteins identified either by our developed method (the top left circle) or by Chen’s protein ranking method (the top right circle), and the number of proteins which have been confirmed to be related with ARVC in literature were plotted. (DOC) [file pone.0071191.s006.doc]
