# Supplementary material for: Prioritizing Disease Candidate Proteins in Cardiomyopathy-Specific Protein-Protein Interaction Networks Based on “Guilt by Association” Analysis
Source: PLoS One. 2013 Aug 5;8(8):e71191. doi: 10.1371/journal.pone.0071191 (PMC3733802; doi:10.1371/journal.pone.0071191)
Supplement: Table S1 — Top 50 candidate proteins from HCM-specific PPIN. (DOC) [file pone.0071191.s007.doc]

**Table S1. Top 50 candidate proteins from HCM-specific PPIN.**

| **Protein****^*^** | **Accession number** | **Rank** | **Disease relevance score** | **Relevance** | **Literature** | **PubMed ID** |
| --- | --- | --- | --- | --- | --- | --- |
| ACTN2 | P35609 | 1 | 34768839.440 | HCM | Diagnostic, prognostic, and therapeutic implications of genetic testing for hypertrophic cardiomyopathy | PMID: 19589432 |
| MYH14 | Q7Z406 | 2 | 34471862.760 |  |  |  |
| TCAP | O15273 | 3 | 31167929.550 | HCM | Echocardiographic-determined septal morphology in Z-disc hypertrophic cardiomyopathy; Genotype-phenotype relationships involving hypertrophic cardiomyopathy-associated mutations in titin, muscle LIM protein, and telethonin | PMID: 17097056; PMID: 16352453 |
| DES | P17661 | 4 | 29448464.290 | cardiomyopathy | Desmin mutation responsible for idiopathic dilated cardiomyopathy; Dual color photoactivation localization microscopy of cardiomyopathy-associated desmin mutants | PMID: 10430757; PMID: 22403400 |
| TNNI1 | P19237 | 5 | 24275542.270 |  |  |  |
| DMD | P11532 | 6 | 22146129.100 | cardiomyopathy | Dystrophin muscle enhancer 1 is implicated in the activation of non-muscle isoforms in the skeletal muscle of patients with X-linked dilated cardiomyopathy; Evidence for a dystrophin missense mutation as a cause of X-linked dilated cardiomyopathy | PMID: 11726549; PMID: 9170407 |
| TNNI2 | P48788 | 7 | 10788905.090 |  |  |  |
| MYL1 | P05976 | 8 | 7530771.846 |  |  |  |
| ACTA1 | P68133 | 9 | 7392504.267 | HCM | Nemaline myopathy and non-fatal hypertrophic cardiomyopathy caused by a novel ACTA1 E239K mutation; Fatal hypertrophic cardiomyopathy and nemaline myopathy associated with ACTA1 K336E mutation | PMID: 21570694; PMID: 16945537 |
| NEB | P20929 | 10 | 6999297.693 |  |  |  |
| TPM2 | P07951 | 11 | 6279716.287 | cardiac dysfunction | Cap disease due to mutation of the beta-tropomyosin gene (TPM2) | PMID: 19345583 |
| TNNC2 | P02585 | 12 | 5488960.917 |  |  |  |
| TNNT3 | P45378 | 13 | 3933672.120 |  |  |  |
| CALM1 | P62158 | 14 | 3435824.468 | cardiomyopathy | Protective action of tetramethylpyrazine phosphate against dilated cardiomyopathy in cTnT(R141W) transgenic mice | PMID: 20154713 |
| TNNT1 | P13805 | 15 | 3332291.074 |  |  |  |
| MYL4 | P12829 | 16 | 2347402.094 |  |  |  |
| DNAH8 | Q96JB1 | 17 | 2232765.898 |  |  |  |
| TMOD1 | P28289 | 18 | 2128568.959 | cardiomyopathy | Genomic organization of mouse and human erythrocyte tropomodulin genes encoding the pointed end capping protein for the actin filaments | PMID: 11054557 |
| TPM4 | P67936 | 19 | 1949939.970 |  |  |  |
| MYH3 | P11055 | 20 | 1563821.009 |  |  |  |
| MYBPC2 | Q14324 | 21 | 1523029.440 |  |  |  |
| TPM3 | P06753 | 22 | 1497133.191 |  |  |  |
| LDB3 | O75112 | 23 | 1428274.029 | HCM | Diagnostic, prognostic, and therapeutic implications of genetic testing for hypertrophic cardiomyopathy | PMID: 19589432 |
| MYL6B | P14649 | 24 | 1343803.687 |  |  |  |
| NKX2-5 | P52952 | 25 | 1337261.499 | cardiomyopathy | Nkx2-5 pathways and congenital heart disease; loss of ventricular myocyte lineage specification leads to progressive cardiomyopathy and complete heart block | PMID: 15109497 |
| MYBPC1 | Q00872 | 26 | 1254247.150 |  |  |  |
| MYL9 | P24844 | 27 | 1223681.865 |  |  |  |
| MYOD1 | P15172 | 28 | 1113322.143 | cardiomyopathy | Demonstration of MyoD1 expression in oncocytic cardiomyopathy: report of two cases and review of the literature | PMID: 15157052 |
| LMNA | P02545 | 29 | 859387.474 | HCM | Cardiac findings in congenital muscular dystrophies | PMID: 20679303 |
| VIM | P08670 | 30 | 836991.861 |  |  |  |
| MYH11 | P35749 | 31 | 789164.308 |  |  |  |
| NPPA | P01160 | 32 | 566003.664 | HCM | In vivo natriuretic peptide reporter assay identifies chemical modifiers of hypertrophic cardiomyopathy signalling | PMID: 22198505 |
| SGCD | Q92629 | 33 | 500947.881 | cardiomyopathy | Mutations in the human delta-sarcoglycan gene in familial and sporadic dilated cardiomyopathy | PMID: 10974018 |
| GJA1 | P17302 | 34 | 496885.782 | cardiac arrhythmias | Cardiac connexins, mutations and arrhythmias | PMID: 22382502 |
| MYL6 | P60660 | 35 | 399682.868 |  |  |  |
| MYLK | Q15746 | 36 | 376102.822 |  |  |  |
| PROC | P04070 | 37 | 309669.182 |  |  |  |
| CKM | P06732 | 38 | 299809.584 | cardiomyopathy | Selective decrease of components of the creatine kinase system and ATP synthase complex in chronic Chagas disease cardiomyopathy | PMID: 21738806 |
| MYOG | P15173 | 39 | 270853.731 |  |  |  |
| ACTB | P60709 | 40 | 241882.333 |  |  |  |
| PIN4 | Q9Y237 | 41 | 230105.984 |  |  |  |
| PIK3CD | O00329 | 42 | 221212.877 |  |  |  |
| MYH10 | P35580 | 43 | 220331.739 |  |  |  |
| PRKCA | P17252 | 44 | 194947.965 |  |  |  |
| MYH2 | Q9UKX2 | 45 | 166295.372 |  |  |  |
| CALD1 | Q05682 | 46 | 163869.411 |  |  |  |
| DNM2 | P50570 | 47 | 159713.924 |  |  |  |
| MYH8 | P13535 | 48 | 138156.415 | cardiac tumorigenesis | Clinical phenotypes and molecular genetic mechanisms of Carney complex; Mutation of perinatal myosin heavy chain associated with a Carney complex variant | PMID: 15992699; PMID: 15282353 |
| MYH1 | P12882 | 49 | 113756.815 |  |  |  |
| GATA4 | P43694 | 50 | 113672.198 | HCM | Altered expression of early cardiac marker genes in circulating cells of patients with hypertrophic cardiomyopathy | PMID: 18005871 |

^*^Proteins are represented in their corresponding gene symbols.
