# Supplementary material for: Prioritizing Disease Candidate Proteins in Cardiomyopathy-Specific Protein-Protein Interaction Networks Based on “Guilt by Association” Analysis
Source: PLoS One. 2013 Aug 5;8(8):e71191. doi: 10.1371/journal.pone.0071191 (PMC3733802; doi:10.1371/journal.pone.0071191)
Supplement: Table S2 — Top 50 candidate proteins from ARVC-specific PPIN. (DOC) [file pone.0071191.s008.doc]

**Table S2. Top 50 candidate proteins from ARVC-specific PPIN.**

| **Protein****^*^** | **Accession number** | **Rank** | **Disease relevance score** | **Relevance** | **Literature** | **PubMed ID** |
| --- | --- | --- | --- | --- | --- | --- |
| ANGEL1 | Q9UNK9 | 1 | 15406.558 |  |  |  |
| DSG1 | Q02413 | 2 | 11729.179 |  |  |  |
| CDH17 | Q12864 | 3 | 6821.896 |  |  |  |
| PKP1 | Q13835 | 4 | 6127.627 |  |  |  |
| CTNNB1 | P35222 | 5 | 5206.454 | cardiomyopathy | Beta-catenin accumulates in intercalated disks of hypertrophic cardiomyopathic hearts | PMID: 14613867 |
| CDH2 | P19022 | 6 | 4246.597 |  |  |  |
| DSC1 | Q08554 | 7 | 3317.328 | cardiac muscle | Desmocollin switching in colorectal cancer | PMID: 17088906 |
| PKP3 | Q9Y446 | 8 | 3156.177 |  |  |  |
| CDH1 | P12830 | 9 | 3035.335 |  |  |  |
| DSG3 | P32926 | 10 | 2449.951 |  |  |  |
| GPNMB | Q14956 | 11 | 1609.201 |  |  |  |
| VASH1 | Q7L8A9 | 12 | 1284.938 |  |  |  |
| GJA1 | P17302 | 13 | 1092.383 | cardiac arrhythmias | Cardiac connexins, mutations and arrhythmias | PMID: 22382502 |
| TJP1 | Q07157 | 14 | 1016.539 |  |  |  |
| OCLN | Q16625 | 15 | 762.365 |  |  |  |
| DSC3 | Q14574 | 16 | 523.703 | cardiac muscle | Desmocollin switching in colorectal cancer | PMID: 17088906 |
| CDSN | Q15517 | 17 | 482.883 |  |  |  |
| CASP3 | P42574 | 18 | 449.671 | cardiomyopathy | Elevated p53 expression is associated with dysregulation of the ubiquitin-proteasome system in dilated cardiomyopathy; Darbepoetin alfa exerts a cardioprotective effect in autoimmune cardiomyopathy via reduction of ER stress and activation of the PI3K/Akt and STAT3 pathways | PMID: 18375498; PMID: 18586265 |
| DES | P17661 | 19 | 264.309 | ARVC | Dual color photoactivation localization microscopy of cardiomyopathy-associated desmin mutants; Desmin mutations as a cause of right ventricular heart failure affect the intercalated disks | PMID: 22403400; PMID: 20423733 |
| PKP4 | Q99569 | 20 | 195.274 |  |  |  |
| FN1 | P02751 | 21 | 182.334 |  |  |  |
| PPP1R13L | Q8WUF5 | 22 | 163.785 |  |  |  |
| EGFR | P00533 | 23 | 153.632 | cardiomyopathy | Epidermal growth factor receptor gene polymorphisms, R497K, but not (CA)n repeat, is associated with dilated cardiomyopathy | PMID: 19265688 |
| CTNNA3 | Q9UI47 | 24 | 147.693 |  |  |  |
| POMT2 | Q9UKY4 | 25 | 145.493 | cardiac abnormalities | New POMT2 mutations causing congenital muscular dystrophy: identification of a founder mutation | PMID: 17634419 |
| SRC | P12931 | 26 | 126.762 | cardiomyopathy | Activation of mitogen-activated protein kinases and p90 ribosomal S6 kinase in failing human hearts with dilated cardiomyopathy | PMID: 11744021 |
| DUT | P33316 | 27 | 111.516 |  |  |  |
| CTNND1 | O60716 | 28 | 95.251 |  |  |  |
| CDH11 | P55287 | 29 | 83.649 |  |  |  |
| CTNNAL1 | Q9UBT7 | 30 | 79.510 |  |  |  |
| CTNNA1 | P35221 | 31 | 78.115 |  |  |  |
| AHNAK | Q09666 | 32 | 71.763 | cardiomyocyte | Ahnak1 is a tuneable modulator of cardiac Ca(v)1.2 calcium channel activity; Ahnak, a new player in beta-adrenergic regulation of the cardiac L-type Ca2+ channel | PMID: 22038483; PMID: 17045254 |
| LEF1 | Q9UJU2 | 33 | 68.964 |  |  |  |
| DNTT | P04053 | 34 | 68.580 |  |  |  |
| VCL | P18206 | 35 | 58.500 | cardiomyopathy | Metavinculin mutations alter actin interaction in dilated cardiomyopathy | PMID: 11815424 |
| CDH5 | P33151 | 36 | 52.032 |  |  |  |
| KRT18 | P05783 | 37 | 48.757 |  |  |  |
| KRT16 | P08779 | 38 | 39.345 |  |  |  |
| CTNNA2 | P26232 | 39 | 37.382 |  |  |  |
| DSPP | Q9NZW4 | 40 | 33.003 |  |  |  |
| MYH14 | Q7Z406 | 41 | 32.359 |  |  |  |
| APC | P25054 | 42 | 30.793 | cardiomyopathy | β-Catenin accumulates in intercalated disks of hypertrophic cardiomyopathic hearts | PMID: 14613867 |
| MYH7 | P12883 | 43 | 26.525 | cardiomyopathy | Coding sequence mutations identified in MYH7, TNNT2, SCN5A, CSRP3, LBD3, and TCAP from 313 patients with familial or idiopathic dilated cardiomyopathy; Genetic basis of hypertrophic cardiomyopathy: from bench to the clinics | PMID: 19412328; PMID: 17916152 |
| EVPL | Q92817 | 44 | 24.130 |  |  |  |
| ITGA6 | P23229 | 45 | 23.825 |  |  |  |
| PPP2R4 | Q15257 | 46 | 21.417 |  |  |  |
| DSG4 | Q86SJ6 | 47 | 20.919 |  |  |  |
| PSEN1 | P49768 | 48 | 19.540 | cardiomyopathy | Mutations of presenilin genes in dilated cardiomyopathy and heart failure | PMID: 17186461 |
| EGF | P01133 | 49 | 17.164 | myocardium | Epidermal growth factor promotes a cardiomyoblastic phenotype in human fetal cardiac myocytes | PMID: 8912716 |
| SMAD5 | Q99717 | 50 | 14.204 |  |  |  |

^*^Proteins are represented in their corresponding gene symbols.
